# Comparison of nested PCR and real time PCR of Herpesvirus infections of central nervous system in HIV patients

**DOI:** 10.1186/1471-2334-4-55

**Published:** 2004-11-30

**Authors:** Lorenzo Drago, Alessandra Lombardi, Elena De Vecchi, Giuseppe Giuliani, Rosaria Bartolone, Maria Rita Gismondo

**Affiliations:** 1 Laboratory of Clinical Microbiology, L. Sacco Teaching Hospital and Department of Clinical Sciences L. Sacco, University of Milan, Via GB Grassi74, 20157 Milan, Italy

## Abstract

**Background:**

Molecular detection of herpesviruses DNA is considered as the reference standard assay for diagnosis of central nervous system infections. In this study nested PCR and real time PCR techniques for detection of Herpes simplex virus type 1 (HSV-1), Cytomegalovirus (CMV) and Epstein-Barr virus (EBV) in cerebrospinal fluid of HIV patients were compared.

**Methods:**

Forty-six, 85 and 145 samples previously resulted positive for HSV-1, CMV and EBV by nested PCR and 150 randomly chosen negative samples among 1181 collected in the period 1996–2003 were retrospectively reassessed in duplicate by real time PCR and nested PCR.

**Results:**

Samples giving positive results for CMV, HSV-1 and EBV with nested PCR were positive also with real time PCR. One of the negative samples resulted positive for HSV and one for EBV. Real time PCR showed comparable sensitivity and specificity vs nested PCR.

**Conclusion:**

Real time PCR proved to be a suitable method for diagnosis of herpesvirus infections in CNS, showing comparable sensitivity and being less time consuming than nested PCR.

## Background

Opportunistic infections as well as tumors and vascular and metabolic disorders are common in HIV-infected patients [1-3]. Generally, opportunistic viral infections are caused by a broad spectrum of different species with similar clinical patterns, especially those affecting the central nervous system (CNS), where differential diagnosis requires simultaneous screening of a wide range of different viruses [4,5]. Moreover, immunodeficiency induced by HIV infection favours reactivation of herpesviruses which could cause important diseases by themselves [6].

Although the rate of CNS complications is relatively low, if compared with the high prevalence of herpesviruses in the population, these viruses represent the most important pathogens associated with viral encephalitis and meningitis [7,8], being cytomegalovirus (CMV) the most frequently identified virus in HIV-positive patients, followed by Epstein-Barr virus (EBV) and Herpes simplex virus type 1 (HSV-1) [9]. CMV, that is often responsible of asymptomatic infections in immunocompetent host, is able to cause serious manifestations such as retinitis, pneumonia and encephalitis in presence of an alteration of immunoresponse, while the recovery of Epstein-Barr virus (EBV) in cerebrospinal fluid (CSF) seems to be a prognostic sign for the development of cerebral tumors in patients with AIDS [10,11]. HSV-1 is the most commonly detected virus in diagnostic laboratory, being cause of a variety of clinical symptoms in different anatomical sites such as skin, lips, oral cavity and, especially in immunocompromised patients, CNS [12].

One-step or nested polymerase chain reaction (PCR) has rapidly replaced immunological assays based on virus specific Ig antibodies in CSF for laboratory diagnosis of Herpesvirus infections, even if serological methods are considered an additional tool for defining clinical diagnosis.

Although nested PCR is considered the method of choice in terms of specificity [9,13,14], some additional aspects should be considered.

In the last years, introduction of real time PCR has markedly increased the ease and the speed in the virology laboratory due to the relevant technology that permits rapid temperature cycling within a close system. Considering the importance of relationship between viral load in CSF and severity and outcome of disease, an additional advantage of Real Time PCR is the capability to perform simultaneous qualitative and quantitative analysis.

Here is reported our experience gained in the diagnosis of herpesvirus infections of the CNS in HIV patients by means of nested PCR and Real Time PCR, which has been recently applied in our laboratory.

## Methods

### CSF samples collection

A total of 1181 CSF samples collected in the period 1996 – 2003 from HIV patients attending at Luigi Sacco Teaching Hospital of Milan (Italy) affected by acute encephalitis or meningitis or encephalopathy or other neurological syndromes were considered.

Particularly, they consisted of 684, 954 and 933 CSF samples previously tested for HSV-1, CMV and EBV, respectively, by means of nested PCR. Of these, all the positive samples and 150 negative samples randomly chosen were retested by means of nested PCR and real time PCR. Each sample was re-extracted and run in duplicate.

CSF positive and negative samples were stored at -80°C until analysis.

Clinical data of patients with positive samples were available only in the 20% of the total. These patients generally recorded meningoencephalitis signs such as central motor or sensory alterations, consciousness loss, seizures defects.

### Nucleic acid extraction

Spin-column based QIAamp Mini Kit (Quiagen, Hilden, Germany) protocol extraction for CSF was used as indicated by the manufacturer.

This procedure allowed for the rapid purification of DNA from 200 μL of CSF and comprised four successive steps carried out using QIAamp Spin Columns in a standard microcentrifuge. Purified DNA was concentrated at a final volume of 20 μL.

### Nested PCR

Nested PCR was carried out in a 50 μl mixture containing 40 μl of first amplification mix (outer primers, buffer, dNTP)-(Amplimedical SpA-Bioline Division-Italy), 2U/μl Taq DNA polymerase (Roche Diagnostics-Germany) and 5 μl of purified DNA. Primer pairs selecting for *glycoprotein D *gene of HSV-1 [15], for the late protein *gp58 *of CMV [16], and for *Bam HI-W *region of EBV [17] are shown in Table 1.

After an initial 2 min denaturation at 94°C, 35 cycles of 94°C for 30 sec, 55°C for 30 sec and 72°C for 30 sec were carried out, followed by a 5 min extension at 72°C using a thermal cycler (Gene Amp PCR System 2400-Applied Biosystem – Monza – Italy). The reaction mixture for the second amplification round was the same as for the first one, except for the "inner" primers used instead of the "outer" primers. In the second amplification round 44 μl of amplification mix and 1 μl of the first amplification round PCR product were used. The thermal cycling was repeated as for the first amplification round but using 30 cycles after the initial 2 min denaturation. Each amplification run contained a negative control, consisting of water and a positive plasmidial control.

Analysis for the PCR products was performed by means of 4 % agarose gel electrophoresis followed by visualization with ethidium bromide (0.4 μg/mL) staining and UV illumination to confirm the expected products.

### Real Time PCR

For diagnostic real time PCR Taq polymerase RT PCR Kit (Amplimedical SpA-Bioline Division-Turin Italy) was used. Target regions for HSV and CMV were the same as in nested PCR, while EBNA-1 gene was amplified for EBV [9] as shown in Table 2..

The RT PCR was performed in 25 μl mixture containing 20 μl of amplification mix (buffer, dNTPs, Taq gold polymerase, Rox passive fluorocrome, primers and MGB Eclipse probe) and 5 μl of purified DNA.

The amplification program included an initial decontamination with uracile N'-glycosilase at 50°C for 2 min, followed by denaturation at 95°C for 10 min and 45 two steps of 15 sec at 95°C and 1 min at 60°C.

The RT PCR products were detected by measuring fluorescence with passive reference dye in Sequence Detection System ABI Prism 7000 (Applied Biosystem). Control threshold (C_t_) values were calculated by determining the point at which the fluorescence exceeded a background limit of 0.04. Each analytical session comprised also a negative control (distilled water).

Quantification was carried out by analysing four positive plasmidial standards at 10^2^, 10^3^, 10^4 ^and 10^5 ^copies/reaction. The standards were obtained by cloning the target amplification product in a plasmid, which was transformed and cultured in *Escherichia coli*. Plasmidic DNA was purified with a commercial kit (Qiagen) and its concentration determined spectrophotometrically. Then, plasmidic DNA was serially diluted in a stabilizing buffer to the final desired concentration. Amounts of copies/mL in each sample were determined by means of a quantification software (Amplimedical), by considering an extraction recovery of 80%.

## Results

### Nested PCR

Of 954 CSFs previously examined for CMV by means of nested PCR, 85 samples resulted positive. Among the 684 CSFs tested for HSV-1, 46 samples were found positive, while, 145 of 933 CSFs tested resulted positive for EBV.

Reassessment of these positives and of 150 negative samples confirmed results previously obtained with the same method, with the exception of one HSV-1 positive sample, which resulted negative when retested, as shown in Table 3.

### Real Time PCR

Results from real time PCR are reported in Table 3. All the samples for which nested PCR gave positive results were confirmed by Real Time-PCR for CMV, HSV-1 and EBV. Of the 150 samples resulted negative by nested PCR, 148 were negative, while 1 sample resulted positive for HSV and 1 for EBV. The positive sample giving negative result when reassessed by nested PCR was negative also by real time PCR.

### Sensitivity and specificity

By considering nested PCR as gold standard [9,13], sensitivity and specificity of real time PCR are described in Table 4. Comparable sensitivity (100%) and specificity (99–100 %) were found for real time PCR in respect to nested PCR.

## Discussion

Introduction of PCR into routine diagnostic has rapidly gained a pivotal role for diagnosis of a wide range of diseases, supplanting, in many cases, other methods, such as the classical serodiagnosis. This is particularly true for diagnosis of herpes virus infections in immunocompromised patients, where diminished or suppressed virus-specific antibody responses do not reflect possible reactivated herpes mediated aethiologies [18].

Real time PCR has represented a further step forward, since it allows for quantitative detection of target DNA in a single sample over a large range, remaining possible qualitative detection. Even if contradictory results have been found [19-21], quantification of DNA could represent an important issue to evaluate the severity and outcome of herpesvirus encephalitis, and it may be also used to monitor the success of antiviral therapy. This strategy has already been used for monitoring of patients at risk for CMV infections, when viral load kinetic patterns are used to identify patients who are more likely to have recurrence of CMV disease after the initiation of therapy, as well as to identify patients needing treatment [22-24]. Moreover, in these cases, use of a highly sensitive assay could be of crucial value.

In the last years several real-time PCR methods have been developed for detection of herpesviruses in different biological specimens [25-27]. Real-time PCR has been well recognized to offer several advantages over nested PCR other than allowing quantification of viral load: it reduces the risk of amplicon contamination, being a close-system, is a safer laboratory protocol by not using ethidium bromide, and it allows a notable reduction of time required for response.

In the present work, we compared a real time PCR panel for detection of herpesvirus DNA in CSF with methods employing nested PCR. Our results indicate an overall agreement between the two methods, as reported by other authors [9,27,28]. Differences between the two assays were observed for HSV-1 and EBV analysis, where one negative sample was found positive by real time PCR.

Since extraction panels and primers for HSV and CMV were uniform for the both types of assays, inhibitors present in the DNA preparations could not explain the different results obtained for these samples. Moreover, being nested PCR generally considered as the gold standard for diagnosis of herpes virus in CSF, we tried to use similar primers, chemistry and amplification conditions in order to limit differences for a better comparison of the performance of the two methods. Thus, discrepancies could be likely attributed to the different detection of amplification products, although real time PCR has been reported to be as sensitive as nested PCR [29]. This suggestion is also supported by the fact that the two patients with CSF negative for HSV-1 and EBV with nested PCR showed clinical syndromes compatible with viral encephalitis and clinically improved with antiviral treatment (data not shown). These data seem to suggest that real time PCR could be more sensitive than nested PCR. Since the limit of detection is generally calculated by using plasmidial DNA, it may be possible that, although molecular sensitivity is reported to be similar for nested PCR and real-time PCR [27,28], some differences may occur for biological specimens. The sample classified positive for HSV-1, which resulted negative when retested with both methods, was one of the oldest in our collection, dating 1996, and it might have degraded over the 7 years storage. From this point of view, development of standardized quality controls might be very helpful [30].

## Conclusions

Data obtained in this study confirms the validity of real-time PCR method for detection of herpesvirus DNA in CSF specimens of HIV patients, being sensitive, rapid and quantitative. Since specific and rapid diagnosis is the main target in the case of CNS infections, real time PCR could be considered the method of choice, due to its high specificity, sensitivity and rapidity, once proper quality controls will be available.

## Competing interest

The company Amplimedical SpA-Bioline Division-Italy will pay the article processing fees for the manuscript.

## Authors' contributions

LD conceived of the study and participated in its design and coordination, AL carried out real time PCR, EDV participated in data analysis and drafted the manuscript, GG performed statistical analysis, RB carried out nested PCR, MRG participated in design and coordination of the study. All authors read and approved the final manuscript.

## Pre-publication history

The pre-publication history for this paper can be accessed here:



## Figures and Tables

**Table 1:** Nucleotide base sequence of primers used in nested PCR

**Virus**	**Region**		**Primer (5'-3')**
HSV-1	GpD	Outer	ATCACGGTAGCCCGGCGCTGTGACA CATACCGGAACGCACCACACAA
		Inner	CCATATCGACCACACCGACGA GGTAGTTGGTCGTTCGCGCTGAA
CMV	MIEA	Outer	AAGCGGCCTCTGATAACCAAGCC AGCACCATCCTCCTCTTCCTCTGG
		Inner	AGTGTGGATGACCTACGGGCCATCG GGTGACACCAGAGAATCAGAGGAGC
EBV	BamH I – W	Outer	GAGACCGAAGTGAAGGCCCT GGTGCCTTCTTAGGAGCTGT
		Inner	GCCAGAGGTAAGTGGACTTTAAT GAGGGGACCCTGAGACGGGT

**Table 2:** Nucleotide base sequence of primersused for real time PCR

**Virus**	**Region**		**Primer (5'-3')**
HSV-1	GpD	Forward	CATACCGGAACGCACCACACAA
		Reverse	CCATATCGACCACACCGACGA
CMV	MIEA	Forward	AAGCGGCCTCTGATAACCAAGCC
		Reverse	AGCACCATCCTCCTCTTCCTCTGG
EBV	EBNA-1	Forward	ATCAGGGCCAAGACATAGAGATG
		Reverse	CCTTTGCAGCCAATGCAACT

**Table 3:** Results from nested PCR and real-time PCR

	**NESTED PCR**		**REAL TIME PCR**	
	
	**POS**	**NEG**	**POS**	**NEG**
**CMV**	85	150	85	150
**HSV-1**	45	150+1^a ^= 151	45+1^b ^= 46	149+1^a ^= 150
**EBV**	145	150	145 +1^b ^= 146	149

^a^: sample resulted positive in previous nested PCR

^b^: samples resulted negative in nested PCR

**Table 4:** Sensitivity and specificity of nested PCR and real-time PCR

	Real-time PCR	
	
	Sensitivity (%)	Specificity (%)
**CMV**	85/85 = 100%	150/150 = 100%
**HSV-1**	45/45 = 100%	150/151 = 99%
**EBV**	145/145 = 100%	149/150 = 99%

## References

[B1] Langford TD, Letendre SL, Larrea GJ, Masliah E (2003). Changing patterns in the neuropathogenesis of HIV during the HAART era. Brain Pathol.

[B2] Wolff AJ, O'Donnell AE (2003). HIV-related pulmonary infections: a review of the recent literature. Curr Opin Pulm Med.

[B3] Thirlwell C, Sarker D, Stebbing J, Bower M (2003). Acquired immunodeficiency syndrome-related lymphoma in the era of highly active antiretroviral therapy. Clin Lymphoma.

[B4] Calvario A, Bozzi A, Scarasciulli M, Ventola C, Seccia R, Stomati D, Brancasi B (2002). Herpes consensus PCR test: a useful diagnostic approach to the screening of viral diseases of the central nervous system. J Clin Virol.

[B5] Studhal M, Hagberg L, Rekabdar E, Bergstrom T (2000). Herpesvirus DNA detection in cerebral spinal fluid: differences in clinical presentation between alpha-, beta-, and gamma-herpesviruses. Scand J Infect Dis.

[B6] Schacker T (2001). The role of HSV in the transmission and progression of HIV. Herpes.

[B7] Wood MJ (1998). Viral infections in neutropenia-current problems and chemotherapeutic control. J Antimicrob Chemother.

[B8] Minjolle S, Arvieux C, Gautier AL, Jusselin I, Thomas R, Michelet C, Colimon R (2002). Detection of herpesvrus genomes by polymerase chain reaction in cerebrospinal fluid and clinical findings. J Clin Virol.

[B9] Aberle SW, Puchhammer-Stöckl E (2002). Diagnosis of herpesvirus infections of the central nervous system. J Clin Virol.

[B10] Maschke M, Kastrup O, Diener HC (2002). CNS manifestations of cytomegalovirus infections: diagnosis and treatment. CNS Drugs.

[B11] Klein R, Mullges W, Bendszus M, Woydt M, Kreipe H, Roggendorf W (1999). Primary intracerebral Hodgkin's disease: report of a case with Epstein-Barr virus association and review of the literature. Am J Surg Pathol.

[B12] Whitley RJ, Fields BN, Knipe DM, Howley PM (1996). Herpes simplex viruses. In Fields virology.

[B13] Cinque P, Vago L, Marenzi R, Giudici B, Weber T, Corradini R, Ceresa D, Lazzarin A, Linde A (1998). Herpes simplex virus infections of the central nervous system in human immunodeficiency virus-infected patients: clinical management by polymerase chain reaction assay of cerebrospinal fluid. Clin Infect Dis.

[B14] Sauerbrei A, Wutzler P (2002). Laboratory diagnosis of central nervous system infections caused by herpesviruses. J Clin Virol.

[B15] Aurelius E, Johansson B, Sköldenberg B, Forsgren M (1993). Encephalitis in immunocompetent patients due to Herpes simplex virus type 1 or 2 as determined by two specific polymerase chain reaction and antibody assays of cerebrospinal fluid. J Med Virol.

[B16] Fenner TE, Garweg J, Hufert FT, Boehnke M, Schmitz H (1991). Diagnosis of cytomegalovirus-induced retinitis in human immunodeficiency virus type 1-infected subjects by using the polymerase chain reaction. J Clin Microbiol.

[B17] Wakefield AJ, Fox JD, Sawyer AM, Taylor JE, Sweenie CH, Smith M, Emery VC, Hudson M, Tedder RS, Pounder RE (1992). Detection of Herpesvirus DNA in the large intestine of patients with ulcerative colitis and Crohn's disease using the nested polymerase chain reaction. J Med Virol.

[B18] Lo CY, Ho KN, Yuen KY, Lui SL, Li FK, Chan TM, Lo WK, Cheng IK (1997). Diagnosing cytomegalovirus disease in CMV seropositive renal allograft recipients: a comparison between the detection of CMV DNAemia by polymerase chain reaction and antigenemia by CMV pp65 assay. Clin Transplant.

[B19] Domingues RB, Lakeman FD, Mayo MS, Whitley RJ (1998). Application of competitive PCR to cerebrospinal fluid samples from patients with herpes simplex encephalitis. J Clin Microbiol.

[B20] Revello MG, Baldanti F, Sarasini A, Zella D, Zavattoni M, Gerna G (1997). Quantitation of herpes simplex virus DNA in cerebrospinal fluid of patients with herpes simplex encephalitis by the polymerase chain reaction. Clin Diagn Virol.

[B21] Wildemann B, Ehrhart K, Storch-Hagenlocher B, Meyding-Lamade U, Steinvorth S, Hacke W, Haas J (1997). Quantitation of herpes simplex virus type 1 DNA in cells of cerebrospinal fluid of patients with herpes simplex virus encephalitis. Neurology.

[B22] Emery VC, Sabin CA, Cope AV, Gor D, Hassan-Walker AF, Griffiths PD (2000). Application of viral-load kinetics to identify patients who develop cytomegalovirus disease after transplantation. Lancet.

[B23] Emery VC, Griffiths PD (2000). Prediction of cytomegalovirus load and resistance patterns after antiviral chemotherapy. Proc Natl Acad Sci.

[B24] Humar AD, Kumar D, Boivin G, Caliendo AM (2002). Cytomegalovirus (CMV) virus load kinetics to predict recurrent disease in solid-organ transplant patients with CMV disease. J Infect Dis.

[B25] Stöcher M, Leb V, Bozic M, Kessler HH, Halwachs-Bauman G, Landt O, Stekel H, Berg J (2003). Parallel detection of five human herpes virus DNAs by a set of real-time polymerase chain reactions in a single run. J Clin Virol.

[B26] Espy MJ, Uhl JR, Mitchell PS, Thorvilson JN, Svien KA, Wold AD, Smith TF (2000). Diagnosis of Herpes simplex virus infections in the clinical laboratory by LightCycler PCR. J Clin Microbiol.

[B27] O'Neill HJ, Wyatt DE, Coyle PV, McCaughey C, Mitchell F (2003). Real-Time nested multiplex PCR for the detection of Herpes simplex virus types 1 and 2 and *Varicella zoster *virus. J Med Virol.

[B28] Kessler HH, Mühlbauer G, Rinner B, Stelzl E, Berger A, Dörr HW, Santner B, Marth E, Rabenau H (2000). Detection of herpes simplex virus DNA by real-time PCR. J Clin Microbiol.

[B29] Weidmann M, Meyer-König U, Hufert FT (2003). Rapid detection of Herpes simplex virus and Varicella-zoster virus infections by real time PCR. J Clin Microbiol.

[B30] Niesters HGM (2004). Molecular and diagnostic clinical virology in real time. Clin Microbiol Infect.

